# Efficacy of Face-to-Face Delivered Cognitive Behavioral Therapy in Improving Health Status of Patients With Insomnia: A Meta-Analysis

**DOI:** 10.3389/fpsyt.2021.798453

**Published:** 2021-12-23

**Authors:** Dawei Xu, Elizabeth Cardell, Simon A. Broadley, Jing Sun

**Affiliations:** ^1^School of Medicine and Dentistry, Griffith University, Gold Coast, QLD, Australia; ^2^Menzies Health Institute Queensland, Griffith University, Gold Coast, QLD, Australia

**Keywords:** cognitive behavioral therapy (CBT), face-to-face, insomnia, quality of sleep, health outcomes

## Abstract

**Background:** Face-to-face cognitive behavioral therapy (CBT) is one of the most widely used non-pharmacological treatment approaches for insomnia. The aim of this study is to assess the efficacy of face-to-face delivered CBT on health outcomes and to evaluate the effect of CBT components as subgroup variables to explain the efficacy of face-to-face delivered CBT on health outcomes in adults over 18 years old with insomnia.

**Methods:** Relevant randomized controlled trial studies published in the past 22 years were searched through the electronic databases. The Physiotherapy Evidence Database (PEDro) scale was used to assess the quality of the 31 included studies. The mean difference and standard deviation of outcome variables and subgroup variables were analyzed using random effect model, and the heterogeneity among the articles was assessed with the *Q*-test and *I*^2^. Egger regression analysis was used to assess publication bias.

**Results:** The meta-analysis showed a significant reduction in Insomnia Severity Index [standardized mean difference (SMD) = −2.56, 95% CI −3.81 to −1.30, *p* < 0.001], Pittsburgh Sleep Quality Index (SMD = −0.96, 95% CI −1.25 to −0.68, *p* < 0.001), sleep onset latency (SMD = −1.31, 95% CI −2.00 to −0.63, *p* < 0.001), wakening after sleep onset (SMD = −1.44, 95% CI −2.14 to −0.74, *p* < 0.001), number of awakenings (SMD = −1.18, 95% CI −2.10 to −0.26, *p* < 0.05), depression (SMD = −1.14, 95% CI −1.85 to −0.42, *p* < 0.01), and fatigue (SMD = −2.23, 95% CI −3.87 to −0.58, *p* < 0.01), and a significant increase in total sleep time (SMD = 0.63, 95% CI 0.28 to 0.98, *p* < 0.001), sleep efficiency (SMD = 1.61, 95% CI 0.92 to 2.29, *p* < 0.001), and physical health (SMD = 0.42, 95% CI 0.08 to 0.76, *p* < 0.05), in the CBT intervention group compared with the control group. There was no significant change in anxiety (SMD = −0.62, 95% CI −1.55 to 0.32, *p* > 0.05) and mental health (SMD = 1.09, 95% CI −0.59 to 2.77, *p* > 0.05) in CBT intervention group compared with control group. Group-delivered studies with larger number of intervention sessions and longer duration of single session provided a larger improvement in sleep quality.

**Conclusion:** Face-to-face delivered CBT is effective in increasing total sleep time, sleep efficiency, and physical health, and reducing Insomnia Severity Index scores, Pittsburgh Sleep Quality Index scores, sleep onset latency, wakening after sleep onset, number of awakenings, depression, anxiety, and fatigue in patients with insomnia. Face-to-face delivered CBT is more effective when delivered through a larger number of sessions with longer duration of each session, and when delivered in groups. Face-to-face CBT is recommended to provide treatment to patients with insomnia in clinical settings.

**Systematic Review Registration:** www.crd.york.ac.uk/prospero/display_record.php?ID=CRD42020200091, identifier: CRD4202020009.

## Introduction

Insomnia is one type of psychiatric disease that influences the quality, timing, and amount of sleep, resulting in fatigue and mental distress (1). Insomnia has a high prevalence across the world (2). It was reported that the global prevalence of insomnia was up to 30% (3). Mentally, insomnia can impair daytime concentration and generate anxious or depressed feels that may lead to or aggravate other psychiatric diseases such as depression and anxiety (4). Physically, a chronic condition of insomnia can increase the risks of a great amount of chronic diseases such as diabetes, cardiovascular diseases, and cancer (5).

Currently, insomnia is treated with pharmacological and non-pharmacological treatments. Cognitive behavioral therapy (CBT) is one of the widely studied non-pharmacological treatment option for insomnia, with marked long-term and short-term effects (6). It is currently the first-line therapy in managing all kinds of psychiatric diseases including depression, anxiety, and insomnia (7). The CBT approach perceives the psychiatric symptoms are underpinned by distorted cognition and related behaviors, and these symptoms could be reduced if the distorted cognition and behaviors are corrected (8). Based on the CBT principles, multiple CBT strategies were developed to change the thinking and behavioral patterns, and different strategies were involved in CBT treatments (9). For patients with insomnia, previous studies indicated that it was common for these patients to have dysfunctional understandings about sleep and worries about falling asleep (10). These thoughts may lead to behavioral changes such as spending more time in bed trying to fall asleep and irregular sleep times, which may make falling asleep more difficult, further providing reinforcement of the dysfunctional understandings and creating a vicious loop that exacerbates the existing insomnia symptoms (10). Applying CBT to these patients can break this loop from multiple directions. Cognitive behavioral therapy has been commonly delivered in a face-to face format, in which patients has face-to-face consultations with the therapists. The consultations can take place between one patient and a therapist individually (individual delivered) or one or more therapists and multiple patients (group delivered). The face-to-face delivery of CBT is commonly used to patients with insomnia because the components of CBT such as alliances building can be easily achieved in the face-to-face therapy mode. Previous studies have shown that CBT provided a similar efficacy in treating insomnia compared with pharmacological treatments (11). Furthermore, it has been found CBT treatment has fewer side effects than sleep medication intake (6).

Existing meta-analysis studies also found that CBT had a significant overall effect on sleep outcomes in patients with insomnia (12, 13), but these studies focused on sleep outcomes only, and subgroup analysis of CBT components was not included. In addition to sleep outcomes (Insomnia Severity Index, Pittsburgh Sleep Quality Index, total sleep time, sleep efficiency, sleep onset latency, wakening after sleep onset, and number of awakenings), health outcomes were considered in this study, including psychiatric diseases (scores of depression and anxiety scales), fatigue (scores of fatigue scales), and quality-of-life related physical and mental health (scores of quality-of-life surveys). By assessing the effect of face-to-face CBT on these outcomes, our study will provide a comprehensive overview of the effect of face-to-face CBT on the health status of insomnia patients from a different perspective. We also conducted subgroup analysis to assess the effects of different CBT treatment designs and components on the sleep and psychiatric disease outcomes and the overall efficacy of face-to-face CBT to help develop a reasonable CBT treatment plan.

## Methods

The procedure of this study was conducted according to the Preferred Reporting Items for Systematic reviews and Meta-Analysis (PRISMA) guidelines (14). The protocol of this study was registered at the International Prospective Register of Systematic Reviews (PROSPERO Registration ID: CRD42020200091, https://www.crd.york.ac.uk/prospero/display_record.php?ID=CRD42020200091).

### Search Strategy

The database search process was completed by two researchers (Xu and Sun) independently. Five electronic databases including PubMed, Scopus, EMBASE, Cochrane Library, and PsycINFO were searched for relevant studies by the two researchers. Gray literatures were acquired from ProQuest central: Dissertations and Thesis. The following Boolean approach for the keywords were used for the search: “insomnia” OR “sleep” AND “cognitive behavioral therapy” OR “cognitive behavior therapy” OR “CBT” AND “face-to-face delivered” OR “group delivered” OR “individual delivered” AND “randomized controlled trial” OR “randomized controlled trial” OR “RCT.” The search results from the defined five electronic databases were exported into the EndNote X9 software, and duplicates across databases were removed. Two researchers then examined the titles and abstracts of the articles and excluded articles that did not focus on insomnia or CBT. The full text of the rest of the articles were examined and selected based on the inclusion and exclusion criteria.

### Study Selection

Full-text assessment of the articles was also completed by two researchers independently. In this study, PICO approach (15) was used to develop the inclusion and exclusion of articles. Only randomized controlled trial studies were included in this study. The studies must be published in English, and the publishing date should be from 1 January 1990 to 31 August 2021, with no restriction in sample size. Articles that were not peer reviewed or that scored 3 or less on the PEDro (16) scale were excluded from the study. If full text of the article was not available online, reasonable attempts were made, such as sending emails directly to the authors to request the full text of the articles.

#### Inclusion Criteria

P: The participants in the studies must be over 18 years in age and be self-reported or diagnosed with insomnia according to the International Classification of Sleep Disorders (ICSD), diagnostic and statistical manual of mental disorders fourth edition (DSM-IV) or fifth edition (DSM-V), or sleep questionnaires such as the Insomnia Severity Index (score > 7) (17), and Pittsburgh Sleep Quality Index (score > 4) (18). Participants should be randomly assigned to CBT intervention groups or control groups.

I: The face-to-face delivered CBT should be applied to the participants as interventions of insomnia. The CBT intervention must utilize at least two of the core components, including sleep restriction, stimulus control, cognitive therapy, and sleep hygiene. The number of other components in the intervention plan is not limited.
C: Participants in the control group in the studies received normal treatment, were placed in a waiting list (waiting list control, WLC), or received placebo treatments that did not provide significant effects on sleep.
O: The studies must report at least one of the primary outcomes required in this meta-analysis study. The primary outcomes include Insomnia Severity Index, Pittsburgh Sleep Quality Index, total sleep time, sleep efficiency, sleep onset latency, wake after sleep onset, and number of awakenings.

#### Exclusion Criteria

P. The participants in the study were diagnosed with other severe psychiatric diseases including schizophrenia, severe depression, and severe bipolar disorders.
I. The intervention group received mindfulness-based intervention or remote-delivered CBT intervention.
C. No proper control groups presented in the study or patients in the control groups received any forms of face-to-face delivered CBT intervention.
O. The study did not provide complete data for the primary outcomes.

### Quality Assessment

Studies passing the full-text assessment underwent a further quality assessment process conducted by two researchers independently using the Physiotherapy Evidence Database (PEDro) scale (16). The PEDro scale defines the quality of the articles by giving scores ranging from 0 to 10. The marking criteria include random allocation of participants, concealed allocation of participants, similar baseline characteristics between intervention and control groups, blinding to all participants, blinding to all therapists in the study, blinding to assessors who assessed the key outcomes of the study, enough outcome measures obtained, participants received intervention or control condition as study designed, presence of between-group comparison analysis, and presence of both point measures and measures of variability. For each of the criteria, 1 point would be given if the study completely met the criteria; otherwise, no point would be given. Articles scoring 3 or less were defined as poor quality articles (19). Scores 4–5 indicated moderate quality studies, scores 6–8 indicated good quality studies, and scores 9–10 indicated excellent quality studies (19).

### Research Outcomes

The primary outcomes in this study were sleep outcomes, including severity of insomnia measured by the Insomnia Severity Index (17), sleep quality measured by the Pittsburgh Sleep Quality Index (18), total sleep time, sleep efficiency, sleep onset latency, wakening after sleep onset, and number of awakenings.

The secondary outcomes in this study included psychiatric disease outcomes, fatigue, and quality-of-life related physical and mental health. The psychiatric disease outcomes included scores of depression scales (20–24) and anxiety scales (25–27). The fatigue outcome was measured by the scores of various fatigue scales (28, 29). The quality-of-life related health outcomes were measured by the scores of the physical health section and mental health section in the short-form quality-of-life surveys (SF-12 and SF-36) (30).

### Data Extraction

The extraction of data was completed by two researchers independently. The third researcher was invited to confirm the data if disagreement occurred between the two researchers. If the article provided results for both post intervention and follow-up, the results in the longer intervention time for follow-up were extracted. Reasonable attempts, such as direct email requests to the authors, were also made to acquire the full datasets of the studies, if possible, when the published articles did not provide enough data.

Characteristics of the included studies and the PEDro scores are summarized in Table 1. The study characteristics include the location where the study was conducted (country), sample size, gender distribution (percentage of females), average age, type of disorder including any comorbid diseases, diagnosis criteria, drugs used in the study, intervention format, presence of manuals, number of intervention sessions, duration of a single session, total treatment time, type of therapist, percentage of participants that completed the treatment, type of control group, outcome variable names, and length of follow-up period.

**Table 1:** Characteristics of included studies.

**References**	**Location**	***N***	**Female, *n* (%)**	**Age**	**PEDro score**	**Disorder**	**Diagnosis**	**Drugs**	**Intervention**	**Delivery format**
Arnedt et al. (34)	USA	17	6 (35%)	46	6	Insomnia, alcohol dependent	ISI ≥8, ICSD, DSM-IV	Not specified	CBT for insomnia for alcohol dependent	Individual
Ayabe et al. (35)	Japan	51	15 (30%)	60	8	Pharmacotherapy-resistant insomnia	ISI ≥8, ICSD, DSM-IV	Benzodiazepine	Add-on cognitive behavioral treatment for insomnia	Individual
Bothelius et al. (36)	Sweden	66	57 (86%)	51	8	Chronic insomnia	Self-report, daytime impairment	Hypnotics	Manual-guided CBT-I delivered by ordinary primary care personnel	Group
Carney et al. (37)	USA	107	68 (73%)	42	5	Insomnia, depression	ISI ≥15, SE, TWT, SCID, HAMD-17	Escitalopram	CBT for insomnia	Individual
Chen et al. (38)	Taiwan	72	42 (58%)	58	6	Sleep disturbance, renal disease	PSQI >5, self-report	Not specified	CBT for insomnia	Group
Currie et al. (39)	Canada	60	18 (30%)	43	5	Insomnia, alcohol dependent	Self-report, SCID	Hypnotics	Brief individual cognitive behavioral interventions for insomnia	Individual
Drake et al. (40)	USA	154	154 (100%)	56	5	Chronic insomnia	DSM-5, ICSD	Not specified	CBT for insomnia	Individual
Ellis et al. (41)	UK	40	Not reported	33	7	Acute insomnia	DSM-5	Not specified	“Single-shot” CBT for insomnia	Individual
Espie et al. (42)	UK	201	137 (68%)	54	7	Persistent insomnia	DSM-IV, ICSD	Not specified	Nurse-administered small-group CBT	Group
Garland et al. (43)	USA	45	43 (96%)	56	6	Insomnia	ICSD	Armodafinil	CBT for insomnia	Individual
Hou et al. (44)	China	98	56 (57%)	53	8	Insomnia, renal disease	PSQI >7, SCL-90	Not specified	Progressive muscle relaxation, sleep-related behavior modification	Group
Jacobs et al. (45)	USA	30	27 (70%)	47	6	Insomnia	ICSD	Hypnotics	CBT + 1 telephone treatment session	Individual
Jansson-Fröjmark et al. (46)	Sweden	32	20 (63%)	56	5	Insomnia, hearing impairment	ICSD	Not specified	CBT for insomnia	Individual
Jungquist et al. (47)	USA	28	24 (86%)	49	5	Insomnia, chronic pain	ICSD	Drugs for pain	CBT for insomnia	Individual
Lovato et al. (48)	Australia	118	63 (53%)	64	6	Insomnia	PSQI, self-report	Not specified	Brief 4-week group-administered CBT for insomnia	Group
Manber et al. (49)	USA	30	17 (61%)	35	7	Insomnia, major depression	DSM-IV, HRSD-17, ICSD	Escitalopram	CBT for insomnia	Individual
McCrae et al. (50)	USA	76	76 (100%)	53	8	Insomnia, chronic pain	ICSD	Antidepressants, drugs for pain	CBT for insomnia	Individual
Morin et al. (51)	USA	78	50 (64%)	65	5	Insomnia	ICSD	Temazepam	CBT (stimulus control, sleep restriction, sleep hygiene, cognitive therapy)	Group
Norell-Clarke et al. (52)	Sweden	64	25 (77%)	52	8	Insomnia, depressive symptom	DSISD, SCID, BDI	Not specified	Group CBT for insomnia	Group
Pigeon et al. (53)	USA	21	14 (67%)	51	5	Insomnia, chronic pain	ICSD, apnea-hypopnea index <10	Not specified	CBT for insomnia, CBT for pain	Individual
Sadler et al. (54)	Australia	72	40 (56%)	75	4	Insomnia, depression	DSM-5	Not specified	CBT for insomnia	Group
Savard et al. (55)	Canada	58	58 (100%)	54	8	Insomnia secondary to cancer	IIS, ICSD	Hypnotics	Immediately delivered CBT	Group
Schiller et al. (56)	Sweden	51	32 (63%)	42	7	Moderate insomnia	Self-report	Not specified	Workplace-based group CBT for insomnia	Group
Sivertsen et al. (57)	Norway	30	16 (53%)	61	5	Chronic insomnia	DSM-IV	Not specified	CBT (sleep hygiene, sleep restriction, stimulus control, cognitive therapy, and relaxation)	Individual
Smith et al. (58)	USA	100	79 (79%)	59	5	Insomnia, osteoarthritis	ICSD, self-report	Not specified	Standardized CBT for insomnia	Individual
Soeffing et al. (59)	USA	47	Not reported	64	8	Insomnia, hypnotic dependent	SCID, ICSD	Hypnotics	CBT for insomnia	Individual
Song et al. (60)	Korea	40	26 (65%)	56	6	Insomnia, restless leg syndrome	ICSD-3	Not specified	CBT for insomnia	Group
Talbot et al. (61)	USA	45	31 (69%)	37	5	Insomnia, PTSD	DSM-IV	Not specified	CBT for insomnia	Individual
Taylor et al. (63)	USA	100	85 (85%)	32	5	Insomnia	DSM-5, SE <85%, self-report	Not specified	CBT for insomnia	Group
Taylor et al. (62)	USA	151	27 (18%)	32	8	Insomnia	DSM-5, SE <85%, self-report	Not specified	CBT for insomnia	Group
Vitiello et al. (64)	USA	367	288 (79%)	73	8	Insomnia, osteoarthritis pain	Self-report	Not specified	CBT for insomnia	Group
**References**		**Manual**	**Number of sessions (** ***n*** **)**	**Duration of sessions (min)**	**Duration of treatment (weeks)**	**Therapist type**	**Compliance (%)**	**Control**	**Outcome measurements**	**Follow up**
Arnedt et al. (34)		Yes	8	15–60	8 w	2 Authors with certificate	59	Behavioral placebo treatment	ISI, sleep diaries, BDI-II, STAI-T, MFI-20, SF-36	N/A
Ayabe et al. (35)		Yes	5	50	10 w	Trained clinical psychologists	96	Treatment as usual	ISI, PSQI, sleep diaries, SDS	4 w
Bothelius et al. (36)		Yes	5	60–90	9 w	4 Primary health-care nurses and 1 social worker	85	Wait list control	ISI, sleep diaries	18 m
Carney et al. (37)		Yes	4	60	8 w	Master's-level students	63	Sleep hygiene	ISI, Sleep diaries, HAMD-17	6 m
Chen et al. (38)		No	2	30	6 w	2 Psychiatrists and 1 assistant psychologist	100	Sleep hygiene	PSQI, sleep diaries, BAI, BDI, FSS	N/A
Currie et al. (39)		Yes	5	60	7 w	3 Mental health professionals	78%	Wait list control	PSQI, sleep diaries, BDI	6 m
Drake et al. (40)		No	6	<60	6 w	1 Registered nurse	97	Sleep hygiene	ISI, sleep diaries	6 m
Ellis et al. (41)		No	1	60–70	1 w	1 Practicing health psychologist and somnologist	100	Wait list control	ISI, sleep diaries	1 m
Espie et al. (42)		No	5	60	5 w	7 Primary care nurses	89	Treatment as usual	PSQI, sleep diaries, SF-36	6 m
Garland et al. (43)		No	7	15–60	7 w	Not reported	83	Medication only	Sleep diaries	N/A
Hou et al. (44)		No	36	20	3 m	Not reported	100	Treatment as usual	PSQI, SCL-90	N/A
Jacobs et al. (45)		No	4	30	6 w	Not reported	93	Placebo treatment	Sleep diaries	12 m
Jansson-Fröjmark et al. (46)		Yes	7	<60	7 w	3 Trained psychologists	94	Wait list control	ISI, sleep diaries, HADS	3 m
Jungquist et al. (47)		Yes	8	30–60	8 w	1 Masters prepared nurse therapist	75	Treatment as usual	ISI, sleep diaries, BDI	N/A
Lovato et al. (48)		No	4	60	4 w	5 Trainee psychologists	97	Wait list control	ISI, sleep diaries, flinders fatigue scale, sleep anticipatory anxiety	3 m
Manber et al. (49)		No	7	60	12 w	2 Licensed clinical psychologists	73	Sleep hygiene	ISI, sleep diaries, HRSD-17	N/A
McCrae et al. (50)		Yes	8	50	8 w	3 Predoctoral students in clinical psychology	72	Wait list control	Sleep diaries, BDI, STAI-T	6 m
Morin et al. (51)		No	8	90	8 w	1 Licensed clinical psychologist or 1 postdoctoral	95	Placebo treatment	Sleep diaries	24 m
Norell-Clarke et al. (52)		Yes	4	120	8 w	1 Licensed psychologist	83	Relaxation training	ISI, sleep diaries, BDI-II	6 m
Pigeon et al. (53)		Yes	10	30–60	10 w	2 Experienced CBT psychologists	100	Wait list control	ISI, sleep diaries, CESD-R, MFI	N/A
Sadler et al. (54)		Yes	8	60–90	8 w	2 Therapists	96	Psychoeducation	ISI, sleep diaries, GDS	3 m
Savard et al. (55)		Yes	8	90	8 w	1 Master-level psychologist	93	Wait list control	ISI, sleep diaries	12 m
Schiller et al. (56)		No	5	120	3 m	1 Trained, certified clinical psychologist	100	Wait list control	ISI, sleep diaries, HADS	3 m
Sivertsen et al. (57)		Yes	6	50	6 w	2 Clinical psychologists	94	Placebo treatment	Sleep diaries	6 m
Smith et al. (58)		Yes	8	45	8 w	Postdoctoral clinical psychology fellows/faculty	91	Behavioral desensitization	ISI, sleep diaries	6 m
Soeffing et al. (59)		No	8	60	8 w	Doctoral students in clinical psychology	100	sham biofeedback treatment	Sleep diaries	N/A
Song et al. (60)		Yes	4	60	4 w	Not reported	63	Sleep hygiene	ISI, sleep diaries, BDI, BAI	3 m
Talbot et al. (61)		Yes	8	60	8 w	Licensed clinical psychologists, certified psychiatrist	93	Wait list control	ISI, PSQI, sleep diaries, BDI	6m
Taylor et al. (63)		No	6	60	6 w	Licensed clinical psychologists, clinical psychology postdoctoral fellows, licensed clinical social worker	86	Minimal contact	ISI, sleep diaries	6 m
Taylor et al. (62)		No	6	60	6 w	Licensed clinical psychologists, clinical psychology postdoctoral fellows, licensed clinical social worker	87	Minimal contact	ISI, sleep diaries, MFI, BDI, BAI	6 m
Vitiello et al. (64)		No	6	90	6 w	1 master's-level family counselor and 1 Ph.D. psychologist	93	Education only control	ISI, SE, GDS	9 m

*BAI, Beck anxiety inventory; BDI, Beck depression inventory; CBT, cognitive behavioral therapy; CESD-R: Center for Epidemiologic Studies Depression Scale-revised; DSISD, Duke structured clinical interview for insomnia; DSM-IV, diagnostic and statistical manual of mental disorders fourth edition; DSM-5, diagnostic and statistical manual of mental disorders fifth edition; FSS, fatigue severity scale; GDS, geriatric depression scale; HADS, hospital anxiety and depression scale; HAMD-17, Hamilton depression rating scale; HRSD-17, 17-item Hamilton rating scale for depression; ICSD, international classification of insomnia; IIS, insomnia interview schedule; ISI, insomnia severity index; MFI, multidimensional fatigue inventory; PSQI, Pittsburgh Sleep Quality Index; PTSD, posttraumatic stress disorder; PEDro, Physiotherapy Evidence Database; RT, relaxation training; SDS, self-rating depression scale; SCID, structured clinical interview for DSM-IV; SCL 90, symptom checklist 90; SE, sleep efficiency; SF-36: 36-item medical outcomes study short-form general health survey; SOL, sleep onset latency; STAI-T, state-trait anxiety inventory-trait subscale; TWT, total wake time; WASO, wake after sleep onset*.

The mean and standard deviation for the primary and secondary outcomes were extracted directly from the results sections, tables, and figures in the studies for both the CBT intervention group and the control group before and after the intervention. The mean difference was calculated by subtracting the mean before intervention from the mean after intervention, and the total pooled mean difference was also calculated. The standardized mean difference (SMD) was calculated by dividing the mean difference by the pooled standard deviation (31).

### Subgroup Data Extraction

The designs and groupings of the subgroup variables were based on the recommended CBT design from the CBT for insomnia guidelines (32) written by Perlis and colleagues. Subgroup information was collected mainly based on the characteristics of intervention delivery and CBT components in each study. The characteristics of intervention were collected from each study and coded into subgroup manually. The subgroups include participant dropout rate (0 for <20% drop out rate and 1 for ≥20% drop out rate), number of treatment sessions (0 for <6 sessions and 1 for ≥6 sessions), duration of treatment sessions (0 for <1 h and 1 for ≥1 h), duration of treatment (0 for <6 weeks and 1 for ≥6 weeks), form of delivery (0 for group-delivered and 1 for individual-delivered), drugs used in the study (0 for no drugs, 1 for hypnotics, and 2 for other drugs including antidepressants and pain drugs), and co-morbid diseases (0 for no co-morbid diseases, 1 for psychiatric diseases, and 2 for chronic diseases). The CBT module component subgroups included treatment rationale, sleep hygiene, relapse prevention, relaxation training, and basic sleep information. The presence of each component was coded as 1, and the absence of certain module was coded as 0.

### Data Analysis

Because of expected heterogenicity, a random effects model, which provided a statistical parameter of the variations among the studies (33), was used to measure the mean difference and SMD of all outcome variables, and the results were presented as forest plots. A SMD between 0.2 and 0.5 indicated a small effect size, between 0.5 and 0.8 a medium effect size, and >0.8 a large effect size. In addition, the 95% confidence interval (CI) and *p*-value were determined for both mean difference and SMD. A *p*-value < 0.05 suggested that the result was statistically significant. The *Q* test and *I*^2^*-*values were used to assess the heterogenicity of the studies. The *I*^2^-value ranges from 0 to 100%. An *I*^2^-value larger than 50% indicates that the study has a high heterogenicity and a subgroup analysis is required to explore the possible causes. In addition, Egger regression analysis was used to assess the potential publication bias among the studies for each of the research outcomes, and the funnel plots were presented. A *p*-value >0.05 indicated that the publication bias for the research outcome was not significant. At the study level, sensitivity analysis was conducted by removing one study at a time vs. all the studies together to identify whether the overall publication bias results for the research outcome were related to any particular study or studies. The software used for data analysis was STATA 17.0.

## Results

### Study Selection

**Figure 1:**
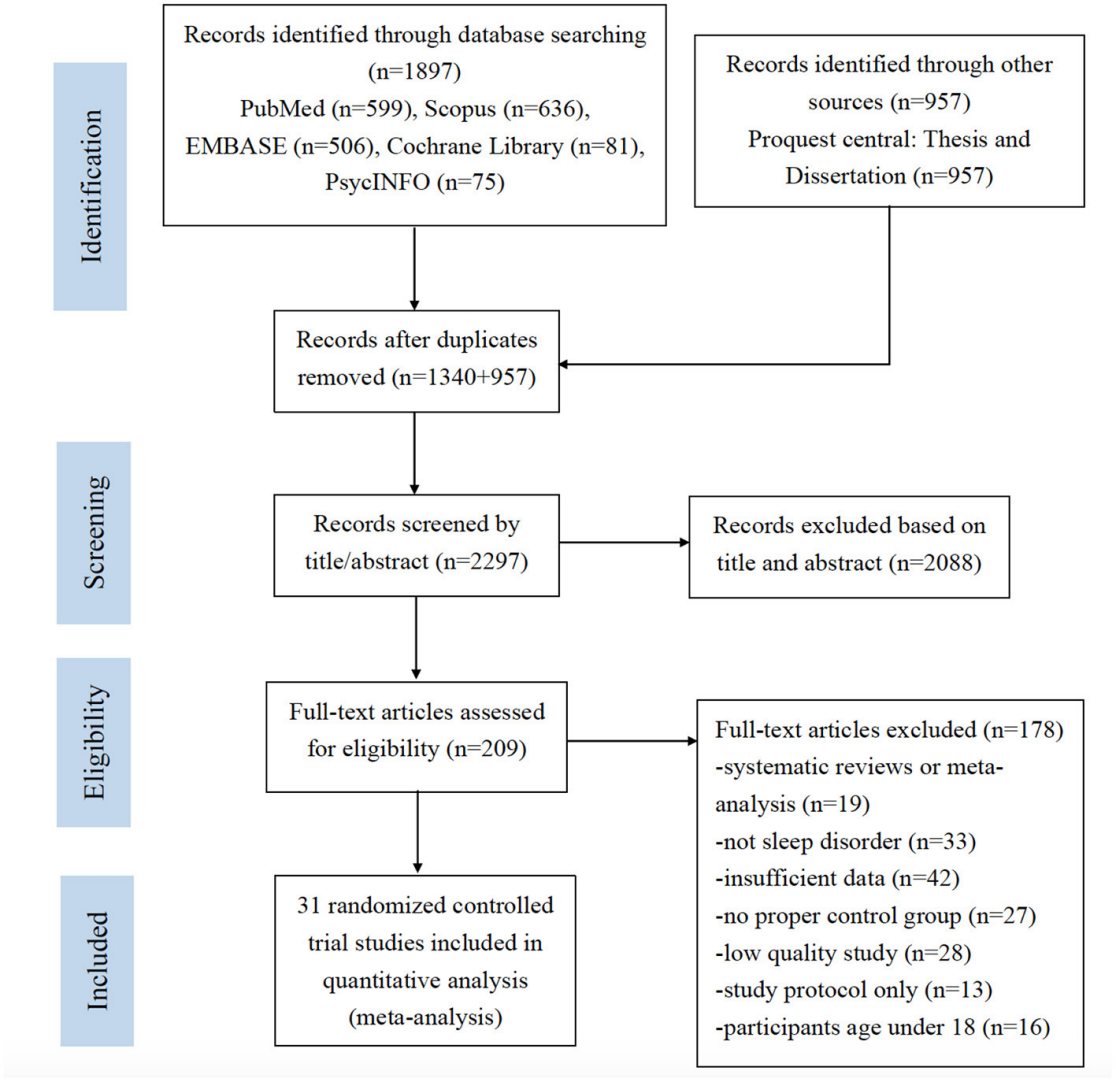
PRISMA flow chart for study selection.

A total of 2,854 studies were identified through database search and other sources. After removing the duplicates, 2,297 studies were screened by titles and abstracts. After the screening of titles and abstracts, 2,088 studies were excluded, and the remaining 209 studies underwent full-text assessment. Another 178 studies were excluded after full-text assessment. Among the 178 excluded studies, 19 were meta-analysis or systematic reviews. Thirty-three studies were not related to insomnia. Forty-two studies did not provide sufficient data for analysis. Twenty-seven studies did not have control groups. Twenty-eight studies provided PEDro scores lower than 3 and were considered as low-quality studies. Thirteen studies were study protocols for clinical trials. Sixteen studies involved participants aged under 18, which did not meet the inclusion criteria. The final number of eligible studies included for meta-analysis was 31. The detailed process of study screening for meta-analysis was presented in Figure 1.

### Study Characteristics

A total number of 2,449 patients were included in the 31 studies (34–64), with 1,107 of which in the intervention group and 1,342 of which in the control group. A total of 12 studies (37, 39, 40, 46, 47, 51, 53, 54, 57, 58, 61, 63) received PEDro scores of 4–5, suggesting that they had moderate quality. The remaining 19 studies (34–36, 38, 41–45, 48–50, 52, 55, 56, 59, 60, 62, 64) received PEDro scores of 6–8, suggesting that they had good quality. The studies were carried out in nine different countries including USA (34, 37, 40, 43, 45, 47, 49–51, 53, 58, 59, 61–64), UK (41, 42), Canada (37, 39, 55), Australia (48, 54), China (38, 44), Japan (35), Sweden (36, 46, 52, 56), Norway (57), and Korea (60). The percentage of female taking part in the studies was 66.9%, and the average age of the participants was 54.6 years. All of the studies included patients with insomnia. Among of which, some of the studies included patients with different co-morbid diseases including alcohol dependence (34, 39), chronic pain (47, 50, 53, 58, 64), renal diseases (38, 44), hearing impairment (46), depression (37, 49, 52, 54), and posttraumatic stress disorder (61). The percentage of patients that reported co-morbid depression was 30.7% (34, 37–39, 46, 47, 49, 50, 52–55, 60–62), and the percentage of patients that reported co-morbid anxiety was 20.1% (34, 38, 46, 50, 54, 55, 60, 62). Thirteen studies (32, 34, 35, 40, 42, 43, 45–47, 49–51, 53, 55) used the ICSD as diagnosis criteria. Six studies (34, 35, 42, 49, 57, 61) diagnosed insomnia using the DSM-IV, while five studies (40, 41, 54, 62, 63) used the DSM-V. All of the included studies used either group-delivered or individual-delivered CBT in the intervention group, with 16 out of 31 studies (34–37, 39, 46, 47, 50, 52–55, 57, 58, 60, 61) using intervention manuals. Moreover, the total number of intervention sessions ranged from 1 to 36 and the time of a single session ranged from 15 to 120 min. The duration of the intervention ranged from 1 day (one single session) to 3 months. The CBT interventions were all delivered by trained professionals. In addition, an average of 88% of the patients completed the treatment across the 31 included studies. All of the included studies reported at least one of the sleep outcomes. The characteristics of studies included for meta-analysis were displayed in Table 1.

### Overall Effect of Face-to-Face CBT on Sleep Outcomes

**Figure 2:**
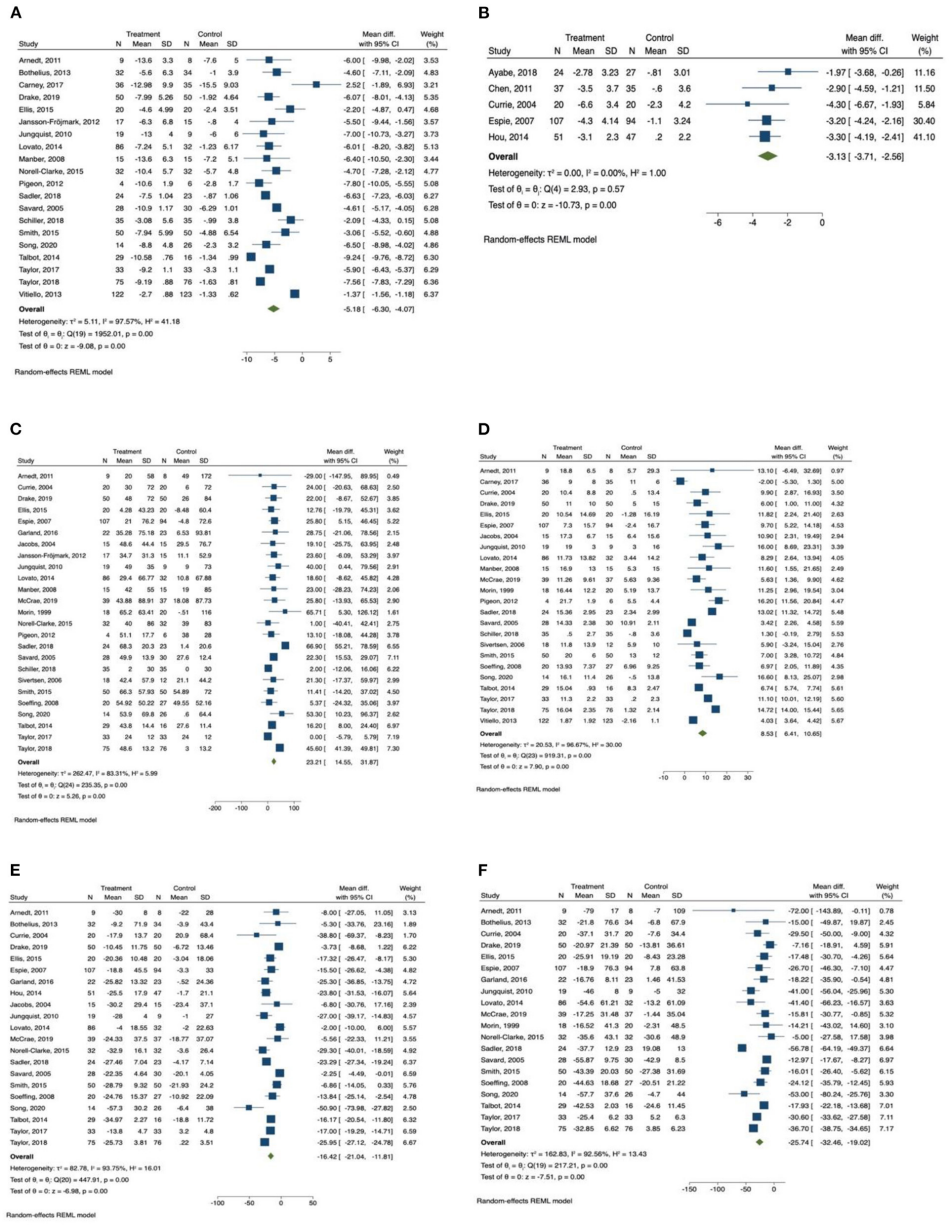
Forest plots of the effect of CBT for sleep outcomes. **(A)** Insomnia Severity Index, **(B)** Pittsburgh Sleep Quality Index, **(C)** total sleep time, **(D)** sleep efficiency, **(E)** sleep onset latency, **(F)** wake after sleep onset.

**Figure 3:**
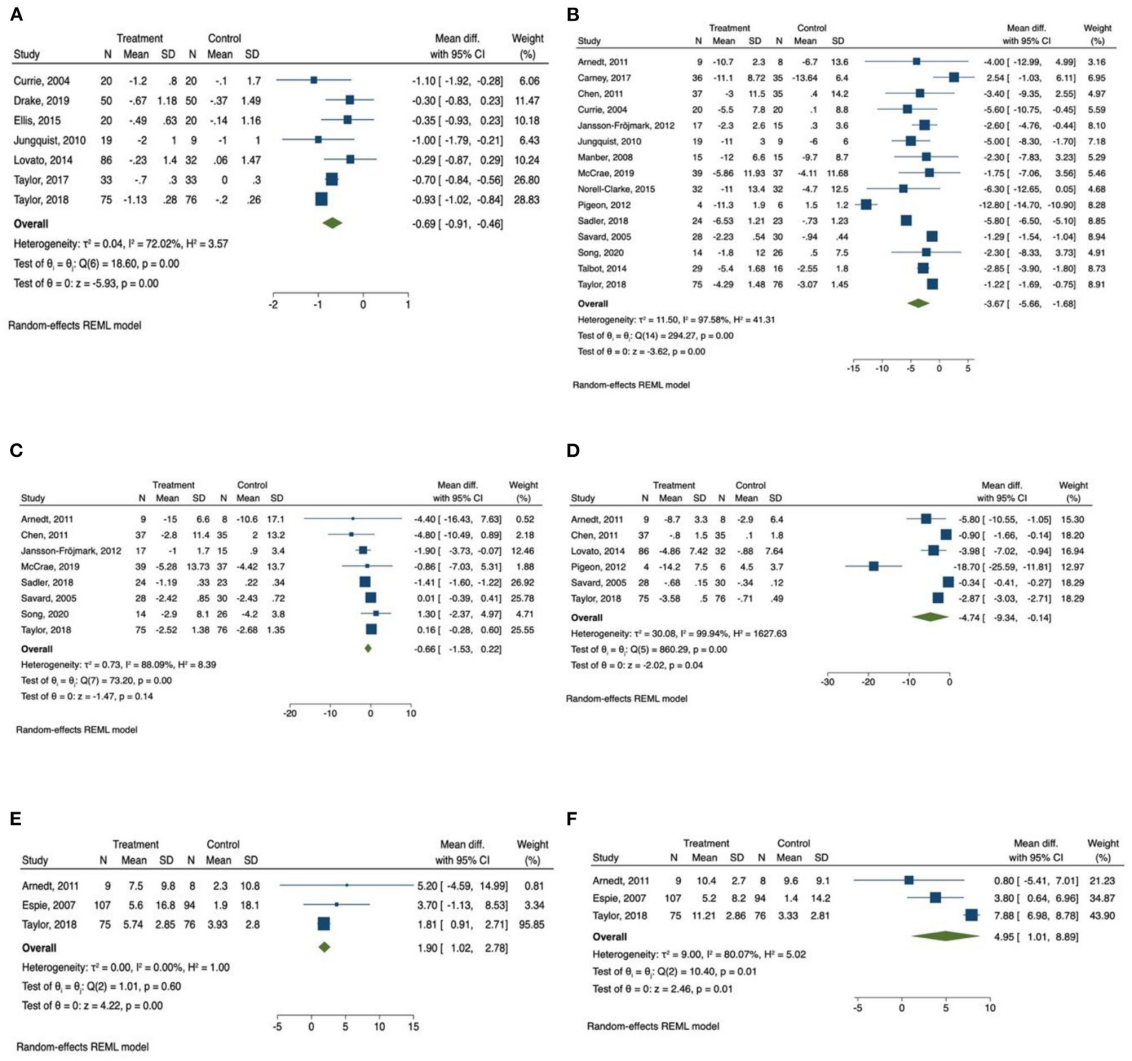
Forest plots of the effect of CBT for sleep and health outcomes. **(A)** Number of awakenings, **(B)** depression, **(C)** anxiety, **(D)** Fatigue, **(E)** physical health, **(F)** mental health.

The results of meta-analysis were presented in Table 2. The forest plots were presented in Figures 2, 3.

**Table 2:** Total effects of CBT on all outcomes.

**Variables**	**Studies (*n*)**	**Participants (*N*)**	**Mean difference**			**Effect size**			**Publication bias**
			**Mean difference (95% CI)**	***Q*-test**	***I*^2^ (%)**	**Standardized mean difference (95% CI)**	***Q*-test**	***I*^2^ (%)**	**Egger's *t* (95% CI)**
Insomnia Severity Index	20	1,398	−5.19^***^ (−6.30, −4.07)	1952.01^***^	97.57^***^	−2.56^***^ (−3.81, −1.30)	516.28^***^	98.89^***^	1.05 (−0.89, 2.69)
Pittsburgh Sleep Quality Index	5	462	−3.13^***^ (−3.71, −2.56)	2.93	0.00	−0.96^***^ (−1.25, −0.68)	7.43	47.62	0.20 (−3.99, 4.52)
Total sleep time	25	1,523	23.21^***^ (14.56, 31.87)	235.35^***^	83.31^***^	0.63^***^ (0.28, 0.98)	215.91^***^	91.20^***^	−0.18 (−1.19, 1.00)
Sleep efficiency	24	1,698	8.53^***^ (6.41, 10.65)	919.31^***^	96.67^***^	1.61^***^ (0.92, 2.29)	474.64^***^	97.26^***^	1.51(−0.36, 2.27)
Sleep onset latency	21	1,477	−16.43^***^ (−21.04, −11.81)	447.91^***^	93.75^***^	−1.31^***^ (−2.00, −0.63)	367.33^***^	97.09^***^	−0.87 (−2.02, 0.84)
Wake after sleep onset	20	1,387	−25.74^***^ (−32.46, −19.02)	217.21^***^	92.56^***^	−1.44^***^ (−2.14, −0.74)	360.23^***^	97.01^***^	−0.42 (−1.76, 1.18)
Number of awakenings	7	543	−0.69^***^ (−0.91, −0.46)	18.60^**^	72.02^**^	−1.18^*^ (−2.10, −0.26)	142.09^***^	95.23^***^	0.98 (−1.22, 2.71)
Depression	15	753	−3.67^***^ (−5.66, −1.68)	294.27^***^	97.58^***^	−1.14^**^ (−1.85, −0.42)	135.21^***^	95.20^***^	0.04 (−1.82, 1.89)
Anxiety	8	493	−0.66 (−1.53, 0.22)	73.20^***^	88.09^***^	−0.62 (−1.55, 0.32)	67.90^***^	95.83^***^	−0.86 (−1.89, 0.90)
Fatigue	6	426	−4.74^*^ (−9.34, −0.14)	860.29^***^	99.94^***^	−2.23^**^ (−3.87, −0.58)	183.37^***^	97.35^***^	−1.77 (−5.01, 1.43)
Physical health	3	369	1.90^***^ (1.02, 2.78)	1.01	0.00	0.42^*^ (0.08, 0.76)	3.85	51.86	1.00 (−9.78, 11.45)
Mental health	3	369	4.95^*^ (1.01, 8.90)	10.40^**^	80.07^**^	1.09 (−0.59, 2.77)	86.20^***^	97.34^***^	−3.18 (−14.87, 8.91)

*CI, confidence interval*.

**#x0002A;:** *p < 0.05*,

** *p < 0.01*,

*** *p < 0.001*.

Insomnia Severity Index was reported by 20 studies (34, 36, 37, 40, 41, 46–49, 52–56, 58, 60–64) as an outcome measurement. Patients in the CBT intervention group showed a significant reduction in insomnia severity as measured by Insomnia Severity Index (mean difference = −5.19, 95% CI −6.30 to −4.07, *p* < 0.001, *I*^2^ = 97.57) compared with the control group, with large effect size (SMD = −2.56, 95% CI −3.81 to −1.30, *p* < 0.001). Subgroup analysis was conducted for Insomnia Severity Index, and the results are presented in Table 3. We found that the subgroup variable that provided greatest effect in improving reducing insomnia severity was relapse prevention. Studies that included relapse prevention (mean difference = −6.765, 95% CI −8.117 to −5.413, *p* < 0.001, *I*^2^ = 84.05) reported a significantly greater improvement in insomnia severity scores than studies that did not include relapse prevention (mean difference = −6.042, 95% CI −7.106 to −4.978, *p* < 0.001, *I*^2^ = 90.56). Other effective subgroup variables included drop-out rate <20%, number of sessions ≥6, duration of one session <1 h, sleep hygiene, and relaxation training.

**Table 3:** Subgroup analysis for Insomnia Severity Index and total sleep time.

**Subgroups**	**Insomnia Severity Index**							**Total sleep time**						
	**Study (*n*)**	**Participant (*n*)**	**MD (95% CI)**	**SMD (95% CI)**	**MD *I*^2^ (%)**	**MD *Q*-test**	***p* Between**	**Study (*n*)**	**Participant (*n*)**	**MD (95% CI)**	**SMD (95% CI)**	**MD *I*^2^ (%)**	**MD *Q*-test**	***p* Between**
Dropout rate <20% ≥20%	15 5	1,194 204	−5.368^***^ (−6.608, −4.128) −4.422^**^ (−7.449, −1.369)	−2.912^***^ (−4.529, −1.295) −1.535^*^ (−2.956, −0.114)	97.93^***^ 79.81^*^	1937.33^***^ 12.81^*^	<0.001	19 6	1,274 249	22.87^***^ (12.66, 33.08) 22.76^***^ (16.31, 29.21)	0.66^**^ (0.23, 1.09) 0.55^*^ (0.03, 1.07)	86.26^***^ 0.00	231.55^***^ 1.50	<0.001
Number of sessions <6 sessions ≥6 sessions	7 13	469 929	−3.691^***^ (−5.616, −1.766) −5.908^***^ (−7.203, −4.612)	−0.718^**^ (−1.152, −0.284) −3.581^***^ (−5.326, −1.836)	73.67^**^ 98.20^***^	20.53^**^ 1931.33^***^	<0.001	8 17	603 920	15.77^**^ (4.12, 27.43) 25.52^***^ (14.51, 36.52)	0.28^**^ (0.11, 0.44) 0.80^**^ (0.30, 1.30)	23.97 88.75^***^	7.97 217.84^***^	<0.001
Duration of one session <1 h ≥1 h	6 14	304 1,094	−6.516^***^ (−8.456, −4.576) −4.668^***^ (−5.965, −3.372)	−2.995 (−6.007, 0.016) −2.399^**^ (−3.777, −1.021)	81.40^**^ 97.94^***^	36.34^***^ 1549.47^***^	<0.001	11 14	532 991	16.40^***^ (9.81, 22.99) 27.18^***^ (14.27, 40.08)	0.32^***^ (0.15, 0.49) 0.85^**^ (0.27, 1.44)	0.00 91.65^***^	2.16 220.71^***^	<0.001
Length of intervention ≤ 6 weeks >6 weeks	7 13	760 638	−5.100^***^ (−6.910, −3.290) −5.223^***^ (−6.712, −3.733)	−2.898^*^ (−5.143, −0.654) −2.368^**^ (−3.937, −0.800)	98.80^***^ 93.47^***^	1443.20^***^ 193.81^***^	<0.001	9 16	776 747	23.47^**^ (9.97, 36.96) 22.99^***^ (11.39, 34.59)	0.67 (−0.02, 1.37) 0.60^**^ (0.22, 0.98)	85.62^***^ 76.93^***^	159.40^***^ 72.19^***^	<0.001
Form of delivery Group–delivered Individual–delivered	10 10	925 473	−5.021^***^ (−6.341, −3.702) −5.304^***^ (−7.311, −3.297)	−3.094^**^ (−4.856, −1.332) −2.009^*^ (−3.826, −0.192)	98.18^***^ 86.56^***^	1535.76^***^ 83.10^***^	<0.001	10 15	853 670	28.04^**^ (12.02, 44.06) 17.10^***^ (10.84, 23.37)	1.03^*^ (0.23, 1.83) 0.33^***^ (0.18, 0.48)	95.15^***^ 0.00	219.32^***^ 3.70	<0.001
Drug treatment No drug used Hypnotics Other	16 1 3	1,203 66 129	−5.384^***^ (−6.556, −4.212) −4.600^***^ (−7.111, −2.089) −3.707 (−9.669, 2.254)	−3.019^***^ (−4.527, −1.510) −0.874^**^ (−1.374, −0.374) −0.707 (−1.760, 0.346)	97.88^***^ N/A 84.43^**^	1939.74^***^ 0.00 12.18^**^	<0.001	17 5 3	1,183 200 140	22.36^***^ (11.95, 32.76) 20.36^*^ (1.50, 39.23) 30.64^*^ (6.05, 55.24)	0.75^**^ (0.25, 1.25) 0.34^*^ (0.06, 0.61) 0.38^*^ (0.04, 0.73)	89.58^***^ 0.00 0.00	231.11^***^ 3.28 0.36	<0.001
Comorbid disease None Psychiatric Chronic	6 9 5	434 736 228	−4.633^***^ (−6.215, −3.052) −5.276^***^ (−7.413, −3.139) −5.410^***^ (−7.142, 3.679)	−0.945^***^ (−1.277, −0.613) −3.924^**^ (−6.448, −1.399) −2.082^**^ (−3.585, −0.579)	64.06^*^ 99.32^***^ 66.78^*^	13.86^*^ 1922.02^***^ 10.65^*^	<0.001	10 8 7	714 460 349	17.44^**^ (6.99, 27.90) 25.70^*^ (5.37, 46.03) 21.99^***^ (15.92, 28.05)	0.31^***^ (0.17, 0.46) 1.03^**^ (0.01, 2.05) 0.59^**^ (0.18, 1.01)	22.46 95.89^***^ 0.00	10.90 207.94^***^ 1.89	<0.001
Treatment rationale No Yes	17 3	1,231 167	−5.480^***^ (−6.614, −4.345) −2.748 (−7.507, 2.011)	−2.929^***^ (−4.351, −1.507) −0.497 (−1.286, 0.293)	97.69^***^ 80.93^*^	1942.46^***^ 8.98^*^	<0.001	22 3	1,351 172	23.79^***^ (14.41, 33.17) 18.59 (−2.04, 39.21)	0.68^**^ (0.29, 1.07) 0.23 (−0.07, 0.53)	85.86^***^ 0.00	233.72^***^ 0.93	<0.001
Sleep hygiene No Yes	4 16	172 1,226	−3.498^**^ (−5.521, −1.476) −5.477^***^ (−6.708, −4.245)	−0.650^***^ (−0.957, −0.343) −3.030^***^ (−4.543, −1.517)	41.70 98.06^***^	5.12 1944.75^***^	<0.001	8 17	325 1,198	15.66^*^ (2.42, 28.90) 24.37^***^ (13.82, 34.92)	0.32^**^ (0.10, 0.53) 0.77^**^ (0.27, 1.27)	18.16 88.83^***^	6.55 219.76^***^	<0.001
Relapse prevention No Yes	12 8	1,076 322	−4.292^***^ (−5.751, −2.833) −6,765^***^ (−8.117, −5.413)	−2.194^**^ (−3.666, −0.722) −3.151^**^ (−5.531, −0.771)	98.25^***^ 84.05^***^	1464.78^***^ 57.67^***^	<0.001	16 9	990 533	19.75^***^ (9.68, 29.81) 28.43^***^ (13.00, 43.87)	0.56^*^ (0.12, 1.00) 0.77^*^ (0.161, 1.37)	81.62^***^ 75.59^***^	181.15^***^ 52.82^***^	<0.001
Relaxation training No Yes	11 9	816 582	−4.472^***^ (−6.296, −2.647) −6.042^***^ (−7.106, −4.978)	−1.997^*^ (−3.623, −0.371) −3.236^**^ (−5.189, −1.282)	97.50^***^ 90.56^***^	71.06 ^***^ 58.29^***^	<0.001	11 14	583 940	19.70^***^ (14.87, 24.53) 25.07^***^ (12.48, 37.65)	0.49^**^ (0.17, 0.81) 0.74^*^ (0.17, 1.32)	0.00 88.72^***^	6.66 215.32^***^	<0.001
Basic sleep information No Yes	10 10	857 541	−5.732^***^ (−7.259, −4.205) −4.511^***^ (−6.153, −2.868)	−4.080^***^ (−6.232, −1.928) −0.869^***^ (−1.267, −0.472)	98.82^***^ 71.68^***^	1920.24^***^ 30.35^***^	<0.001	13 12	764 759	24.91^***^ (12.01, 37.82) 17.93^***^ (8.12, 27.74)	0.89^**^ (0.25, 1.54) 0.31^***^ (0.17, 0.46)	92.27^***^ 18.20	215.45^***^ 11.44	<0.001

*MD, mean difference; SMD, standardized mean difference; CI, confidence interval*.

* *p < 0.05*,

** *p < 0.01*,

*** *p < 0.001*.

Pittsburgh Sleep Quality Index was reported in only five studies (35, 38, 39, 42, 44), and there was an overall significant improvement in sleep quality measured by Pittsburgh Sleep Quality Index in the intervention group (mean difference = −3.13, 95% CI −3.71 to −2.56, *p* < 0.001, *I*^2^ = 0.00) in comparison with the control group, with large effect size (SMD = −0.96, 95% CI −1.25 to −0.68, *p* < 0.001).

Among the sleep quality outcomes, the intervention group reported a significant increase in total sleep time (mean difference = 23.21, 95% CI 14.56 to 31.87, *p* < 0.001, *I*^2^ = 83.31) compared with the control group, with medium effect size (SMD = 0.63, 95% CI 0.28 to 0.98, *p* < 0.001). The results of subgroup analysis for total sleep time are displayed in Table 3. The duration of intervention sessions showed greatest effect on total sleep time. In studies in which the duration of one session was longer than 60 min (mean difference = 27.18, 95% CI 14.27 to 40.08, *p* < 0.001, *I*^2^ = 91.65), a greater effect of CBT was observed than in studies providing sessions shorter than 60 min (mean difference = 16.40, 95% CI 9.81 to 22.99, *p* < 0.001, *I*^2^ = 0.00). Other effective CBT characteristics included number of sessions ≥6, group delivered, treatment with drugs, sleep hygiene, relaxation training, and relapse prevention.

We also found that there was a significant increase in sleep efficiency (mean difference = 8.53, 95% CI 6.41 to 10.65, *p* < 0.001, *I*^2^ = 96.67) in the CBT intervention group compared with the control group, with large effect size (SMD = 1.61, 95% CI 0.92 to 2.29, *p* < 0.001). Subgroup analysis was conducted for sleep efficiency, and the results are presented in Table 4. Both relapse prevention and relaxation training showed a significant effect in improving sleep efficiency. Studies that included relapse prevention (mean difference = 10.834, 95% CI 7.572 to 14.097, *p* < 0.001, *I*^2^ = 87.34) had a greater effect on sleep efficiency than studies that did not include relapse prevention (mean difference = 7.476, 95% CI 4.920 to 10.031, *p* < 0.001, *I*^2^ = 97.01). Likewise, studies that included relaxation training reported a more significant increase in sleep efficiency (mean difference = 9.779, 95% CI 7.087 to 12.472, *p* < 0.001, *I*^2^ = 93.96) than studies that did not include relaxation training (mean difference = 6.787, 95% CI 3.737 to 9.837, *p* < 0.001, *I*^2^ = 95.83). Sleep efficiency was also significantly improved when drop-out rate was <20%, number of sessions were ≥6, length of intervention was ≤ 6 weeks, and sessions were group delivered.

**Table 4:** Subgroup analysis for sleep efficiency and sleep onset latency.

**Subgroups**	**Sleep efficiency**							**Sleep onset latency**						
	**Study (*n*)**	**Participant (*n*)**	**MD (95% CI)**	**SMD (95% CI)**	**MD *I*^2^ (%)**	**MD *Q*-test**	***p* Between**	**Study (*n*)**	**Participant (*n*)**	**MD (95% CI)**	**SMD (95% CI)**	**MD *I*^2^ (%)**	**MD *Q*-test**	***p* Between**
Dropout rate <20% ≥20%	17 7	1,378 320	9.196^***^ (6.953, 11.438) 6.863^**^ (1.908, 11.818)	1.951^***^ (1.032, 2.871) 0.787^**^ (0.286, 1.288)	96.62^***^ 87.45^***^	850.68^***^ 29.50^***^	<0.001	16 5	1,258 219	−17.288^***^ (−22.149, −12.428) −13.494^*^ (−25.890, −1.097)	−1.509^***^ (−2.379, −0.640) −0.634^**^ (−1.101, −0.166)	92.64^***^ 77.70^***^	177.91^***^ 20.90^***^	<0.001
Number of sessions <6 sessions ≥6 sessions	8 16	610 1,088	7.431^**^ (3.017, 11.845) 9.129^***^ (6.820, 11.438)	0.585^***^ (0.281, 0.889) 2.128^***^ (1.180, 3.075)	86.02^***^ 96.90^***^	45.70^***^ 830.25^***^	<0.001	8 13	599 878	−19.335^***^ (−30.200, −8.471) −15.535^***^ (−20.706, −10.365)	−0.643^***^ (−1.023, −0.263) −1.721^***^ (−2.766, −0.676)	78.26^***^ 95.30^***^	29.72^***^ 415.05^***^	<0.001
Duration of one session <1 h ≥1 h	9 15	455 1,243	8.072^***^ (5.567, 10.578) 8.815^***^ (5.841, 11.789)	1.242^**^ (0.406, 2.079) 1.794^***^ (0.831, 2.757)	63.33^*^ 98.11^***^	17.09^*^ 901.29^***^	<0.001	9 12	558 919	−12.918^***^ (−18.726, −7.109) −19.410^***^ (−26.392, −12.428)	−0.732^***^ (−1.157, −0.307) −1.750^**^ (−2.869, −0.631)	73.25^***^ 96.76^***^	31.18^***^ 384.18^***^	<0.001
Length of intervention ≤ 6 weeks >6 weeks	10 14	1,021 677	9.701^***^ (6.909, 12.494) 7.792^***^ (4.770, 10.814)	1.878^**^ (0.572, 3.183) 1.384^***^ (0.664, 2.104)	96.24^***^ 94.48^***^	729.98^***^ 177.78^***^	<0.001	8 13	746 731	−15.975^***^ (−24.535, −7.414) −16.870^***^ (−22.591, −11.149)	−1.747^*^ (−3.406, −0.088) −1.032^***^ (−1.500, −0.565)	96.63^***^ 86.39^***^	145.86^***^ 138.64^***^	<0.001
Form of delivery Group–delivered Individual–delivered	10 14	1,034 664	8.915^***^ (5.658, 12.172) 8.185^***^ (5.317, 11.053)	2.319^**^ (0.995, 3.644) 1.021^***^ (0.484, 1.557)	98.53^***^ 80.33^***^	865.70^***^ 53.59^***^	<0.001	10 11	909 568	−18.678^***^ (−26.363, −10.992) −14.012^***^ (−19.398, −8.625)	−1.862^**^ (−3.198, −0.526) −0.793^***^ (−1.173, −0.412)	97.53^***^ 68.15^***^	382.00^***^ 32.32^***^	<0.001
Drug treatment No drug used Hypnotics Other	16 4 4	1,338 155 205	8.801^***^ (6.334, 11.267) 8.920^***^ (5.581, 12.259) 7.139 (−0.789, 15.066)	2.052^***^ (1.085, 3.019) 0.844^***^ (0.520, 1.167) 0.639 (−0.130, 1.409)	97.60^***^ 0.00 88.28^***^	882.33^***^ 1.19 25.25^***^	<0.001	14 5 2	1,140 233 104	−16.076^***^ (−21.724, −10.427) −18.240^***^ (−27.033, −9.447) −17.089 (−38.040, 3.862)	−1.634^***^ (−2.610, −0.658) −0.588^**^ (−1.010, −0.167) −0.879 (−2.405, 0.648)	96.28^***^ 21.52 75.68^*^	438.32^***^ 5.42 4.11^*^	<0.001
Comorbid disease None Psychiatric Chronic	10 9 5	714 712 272	7.901^***^ (4.943, 10.859) 8.639^***^ (4.754, 12.524) 9.149^**^ (3.953, 14.345)	0.631^***^ (0.478, 0.783) 2.682^**^ (1.148, 4.215) 1.343^***^ (0.591, 2.095)	66.28^***^ 98.86^***^ 90.59^***^	39.63^***^ 800.55^***^ 39.13^***^	<0.001	8 7 6	642 430 405	−12.726^**^ (−21.113, −4.338) −21.382^***^ (−25.870, −16.895) −14.774^**^ (−24.092, −5.455)	−0.482^**^ (−0.781, −0.182) −2.637^**^ (−4.317, −0.958) −0.807^***^ (−1.267, −0.347)	77.14^**^ 87.73^***^ 88.72^***^	25.06^**^ 63.11^***^ 53.07^***^	<0.001
Treatment rationale No Yes	22 2	1,551 147	9.194^***^ (7.141, 11.247) 1.690 (−5.784, 9.163)	1.746^***^ (1.024, 2.468) 0.155 (−0.694, 1.004)	96.19^***^ 86.99^**^	892.26^***^ 7.69^**^	<0.001	19 2	1,337 140	−16.140^***^ (−20.911, −11.369) −18.343 (−41.539, 4.853)	−1.377^***^ (−2.124, −0.630) −0.726 (−1.878, 0.427)	94.10^***^ 81.70^*^	442.00^***^ 5.47^*^	<0.001
Sleep hygiene No Yes	6 18	248 1,450	8.307^***^ (3.654, 12.960) 8.590^***^ (6.156, 11.024)	0.703^***^ (0.450, 0.955) 1.910^***^ (1.022, 2.797)	65.73^**^ 97.47^***^	21.48^**^ 863.02^***^	<0.001	5 16	253 1,224	−21.756^***^ (−26.820, −16.693) −15.184^***^ (−20.628, −9.739)	−0.957^***^ (−1.316, −0.597) −1.443^**^ (−2.334, −0.552)	0.00 95.59^***^	4.22 442.81	<0.001
Relapse prevention No Yes	17 7	1,261 437	7.476^***^ (4.920, 10.031) 10.834^***^ (7.572, 14.097)	1.386^**^ (0.573, 2.198) 2.180^**^ (0.891, 3.469)	97.01^***^87.34^***^	837.06^***^ 55.92^***^	<0.001	14 7	950 527	−15.303^***^ (−21.471, −9.135) −19.871^***^ (−24.995, −14.746)	−1.337^**^ (−2.290, −0.384) −1.254^**^ (−2.117, −0.391)	95.89^***^ 56.53^*^	433.40^***^ 14.13^*^	<0.001
Relaxation training No Yes	11 13	790 908	6.787^***^(3.737, 9.837) 9.779^***^(7.087, 12.472)	1.228^***^(0.563, 1.893) 1.946^**^(0.805, 3.088)	95.83^***^ 93.96^***^	62.51^***^ 282.25^***^	<0.001	9 12	515 962	−14.383^***^ (−21.427, −7.340) −18.259^***^ (−24.314, −12.204)	−0.963^***^ (−1.419, −0.507) −1.564^**^ (−2.721, −0.406)	89.41^***^ 93.10^***^	77.27^***^ 127.32^***^	<0.001
Basic sleep information No Yes	13 11	964 734	8.744^***^ (6.270, 11.218) 8.431^***^ (4.691, 12.171)	2.244^***^ (1.154, 3.334) 0.649^***^ (0.384, 0.914)	97.32^***^ 85.66^***^	813.36^***^ 79.70^***^	<0.001	13 8	832 645	−15.742^***^ (−20.939, −10.544) −18.835^**^ (−29.656, −8.014)	−1.727^**^ (−2.771, −0.683) −0.625^**^ (−1.006, −0.244)	95.25^***^ 79.58^***^	413.47^***^ 30.51^***^	<0.001

*MD, mean difference; SMD, standardized mean difference; CI, confidence interval*.

* *p < 0.05*,

** *p < 0.01*,

*** *p < 0.001*.

In addition, sleep onset latency time was significantly improved in the CBT intervention group (mean difference = −16.43, 95% CI −21.04 to −11.81, *p* < 0.001, *I*^2^ = 93.75) in comparison with the control group, with large effect size (SMD = −1.31, 95% CI −2.00 to −0.63, *p* < 0.001). Subgroup analysis was also conducted for sleep onset latency, and the results are presented in Table 4. We found that the subgroup variable that provided greatest effect in improving sleep onset latency was comorbid disease. There was a significantly greater improvement in sleep onset latency when patients had comorbid psychiatric diseases (mean difference = −21.382, 95% CI −25.870 to −16.895, *p* < 0.001, *I*^2^ = 87.73) compared with patients who had comorbid chronic diseases (mean difference = −14.774, 95% CI −24.092 to −5.455, *p* < 0.01, *I*^2^ = 88.72) and patients who had no comorbid diseases (mean difference = −12.726, 95% CI −21.113 to −4.338, *p* < 0.01, *I*^2^ = 77.14). Other effective CBT characteristics and components included drop-out rate <20%, number of sessions <6, duration of one session ≥60 min, group delivered, relapse prevention, relaxation training, and basic sleep information.

Wake time after sleep onset was also significantly improved in the CBT intervention group (mean difference = −25.74, 95% CI −32.46 to −19.02, *p* < 0.001, *I*^2^ = 92.56) compared with the control group, with large effect size (SMD = −1.44, 95% CI −2.14 to −0.74, *p* < 0.001). The results of subgroup analysis for wakening after sleep onset are displayed in Table 5. The subgroup variables that provided greatest effect in improving wake time after sleep onset were duration of intervention session and relaxation training. The subgroup analysis indicated that wakening after sleep onset was more significantly improved in studies providing intervention sessions longer than 1 h (mean difference = −30.149, 95% CI −39.022 to −21.296, *p* < 0.001, *I*^2^ = 94.17) compared with studies providing sessions shorter than 1 h (mean difference = −17.375, 95% CI −20.768 to −13.987, *p* < 0.001, *I*^2^ = 0.00). A greater improvement in wakening after sleep onset was observed in studies that included relaxation training (mean difference = −30.034, 95% CI −40.118 to −19.950, *p* < 0.001, *I*^2^ = 94.89) compared with studies that did not include relaxation training (mean difference = −19.597, 95% CI −25.892 to −13.302, *p* < 0.001, *I*^2^ = 62.68). Other effective CBT characteristics and components included group delivered, comorbid psychiatric diseases, sleep hygiene, and relapse prevention.

**Table 5:** Subgroup analysis for wake after sleep onset and depression.

**Subgroups**	**Wake after sleep onset**							**Depression**						
	**Study (*n*)**	**Participant (*n*)**	**MD (95% CI)**	**SMD (95% CI)**	**MD *I*^2^ (%)**	**MD *Q*–test**	***p* Between**	**Study (*n*)**	**Participant (*n*)**	**MD (95% CI)**	**SMD (95% CI)**	**MD *I*^2^ (%)**	**MD *Q*-test**	***p* Between**
Dropout rate <20% ≥20%	15 5	1,168 219	−25.810^***^ (−33.770, −17.850) −25.133^***^ (−38.650, −11.615)	−1.549^**^ (−2.475, −0.623) −1.086^***^ (−1.610, −0.562)	93.85^***^ 73.45^**^	152.11^***^ 16.20^**^	<0.001	8 7	461 292	−4.725^**^ (−7.598, −1.853) −2.087 (−4.313, 0.138)	−1.726^*^ (−3.163, −0.289) −0.686 (−1.398, 0.026)	97.44^***^ 62.28	221.19^***^ 12.46	<0.001
Number of sessions <6 sessions ≥6 sessions	7 13	569 818	−25.781^***^ (−36.605, −14.956) −25.564^***^ (−34.024, −17.104)	−0.545^***^ (−0.806, −0.283) −1.909^***^ (−2.917, −0.902)	42.04 95.61^***^	10.49 204.31^***^	<0.001	5 10	287 466	−2.571 (−6.156, 1.013) −4.055^**^ (−6.467, −1.643)	−0.239 (−0.580, 0.102) −1.692^**^ (−2.787, −0.598)	56.75^*^ 98.52^***^	10.00^*^ 284.23^***^	<0.001
Duration of one session <1 h ≥1 h	7 13	430 957	−17.375^***^ (−20.762, −13.987) −30.149^***^ (−39.002, −21.296)	−0.880^**^ (−1.414, −0.346) −1.735^**^ (−2.764, −0.705)	0.00 94.17^***^	6.59 142.31^***^	<0.001	6 9	252 501	−4.830^*^ (−8.699, −0.961) −2.813^**^ (−4.777, −0.850)	−1.217 (−2.545, 0.112) −1.136^*^ (−2.095, −0.178)	92.17^***^ 96.80^***^	86.79^***^ 160.42^***^	<0.001
Length of intervention ≤ 6 weeks >6 weeks	7 13	716 671	−28.502^***^ (−38.685, −18.318) −23.996^***^ (−32.979, −15.012)	−1.956^*^ (−3.632, −0.280) −1.147^***^ (−1.747, −0.546)	93.85^***^ 87.67^***^	40.32^***^ 115.79^***^	<0.001	3 12	263 490	−1.240^***^ (−1.704, −0.775) −4.031^**^ (−6.391, −1.671)	−0.494^*^ (−0.912, −0.077) −1.374^**^ (−2.320, −0.429)	0.00 96.87^***^	0.63 284.44^***^	<0.001
Form of delivery Group-delivered Individual-delivered	10 10	849 538	−30.329^***^ (−41.395, −19.263) −19.934^***^ (−25.494, −14.374)	−1.887^**^ (−3.213, −0.560) −0.963^***^ (−1.386, −0.539)	96.17^***^ 47.72	135.77^***^ 16.72	<0.001	6 9	432 321	−3.047^**^ (−5.187, −0.908) −3.955^*^ (−7.009, −0.901)	−1.468^*^ (−2.841, −0.095) −0.707^**^ (−1.231, −0.184)	97.37^***^ 90.25^***^	149.21^***^ 102.55^***^	<0.001
Drug treatment No drug used Hypnotics Other	13 5 2	1,047 236 104	−26.880^***^ (−36.232, −17.529) −22.412^***^ (−30.592, −14.233) −28.393^*^ (−53.079, −3.708)	−1.795^**^ (−2.820, −0.771) −0.607^**^ (−0.965, −0.249) −1.230 (−2.823, 0.363)	96.23^***^ 0.00 81.54^*^	206.58^***^ 1.24 5.42^*^	<0.001	10 1 4	508 40 205	−4.259^**^ (−6.761, −1.757) −5.600^*^ (−10.754, −0.446) −1.605 (−5.165, 1.955)	−1.630^**^ (−2.744, −0.516) −0.660^*^ (−1.285, −0.036) −0.248 (−0.811, 0.315)	98.55 N/A 63.86	282.91^***^ 0.00 9.32^*^	<0.001
Comorbid disease None Psychiatric Chronic	8 7 5	650 430 307	−22.820^***^ (−32.381, −13.258) −31.968^***^ (−45.169, −18.766) −19.743^***^ (−29.190, −10.295)	−0.565^***^ (−0.819, −0.311) −2.736^**^ (−4.381, −1.090) −0.949^***^ (−1.482, −0.415)	52.33^*^ 97.58^***^ 71.19^*^	14.37^*^ 109.84^***^ 12.26^*^	<0.001	1 8 6	40 276 437	−2.300 (−8.335, 3.735) −2.922^**^ (−5.084, −0.759) −4.623^*^ (−8.368, −0.877)	−0.243 (−0.882, 0.396) −1.036^*^ (−2.049, −0.023) −1.627^*^ (−3.192, −0.061)	N/A 93.67^***^ 94.80^***^	0.00 125.19^***^ 144.30^***^	<0.001
Treatment rationale No Yes	18 2	1,247 140	−27.238^***^ (−34.324, −20.152) −12.513^*^ (−24.985, −0.041)	−1.574^***^ (−2.334, −0.814) −0.300 (−0.655, 0.056)	93.22^***^ 0.00	208.59^***^ 0.61	<0.001	11 4	510 243	−4.341^***^ (−6.656, −2.025) −1.594 (−4.900, 1.712)	−1.532^**^ (−2.525, −0.539) −0.238 (−0.695, 0.220)	98.22 63.66	286.07^***^ 8.11^*^	<0.001
Sleep hygiene No Yes	4 16	163 1,224	−19.641^***^ (−28.584, −10.699) −27.058^***^ (−35.085, −19.031)	−0.640^***^ (−0.950, −0.330) −1.647^***^ (−2.505, −0.790)	0.00 94.83^***^	1.15 210.35^***^	<0.001	3 12	102 651	−2.961^**^ (−4.834, −1.089) −3.726^**^ (−6.133, −1.319)	−0.593^**^ (−0.982, −0.204) −1.337^**^ (−2.295, −0.380)	0.00 98.39	1.17 291.81	<0.001
Relapse prevention No Yes	13 7	860 527	−24.200^***^ (−31.025, −17.375) −27.126^***^ (−41.376, −12.876)	−1.469^**^ (−2.387, −0.550) −1.391^*^ (−2.540, −0.241)	89.18^***^ 90.08^***^	123.21^***^ 89.22^***^	<0.001	7 8	449 304	−1.273^***^ (−1.495, −1.052) −5.120^***^ (−7.856, −2.384)	−0.654 (−1.329, 0.021) −1.765^*^ (−3.204, −0.327)	0.00 93.81^***^	8.12 91.71^***^	<0.001
Relaxation training No Yes	10 10	553 834	−19.597^***^ (−25.892, −13.302) −30.034^***^ (−40.118, −19.950)	−0.949^***^ (−1.404, −0.495) −1.912^**^ (−3.221, −0.604)	62.68^*^ 94.89^***^	19.93^*^ 79.11^***^	<0.001	7 8	313 440	−2.239^*^ (−4.182, −0.296) −4.651^**^ (−7.616, −1.686)	−0.877^*^ (−1.616, −0.139) −1.569^*^ (−3.059, −0.080)	83.57^**^ 96.81^***^	19.99^**^ 221.23^***^	<0.001
Basic sleep information No Yes	11 9	704 683	−27.019^***^ (−36.458, −17.579) −22.827^***^ (−30.934, −14.721)	−2.192^***^ (−3.312, −1.072) −0.497^***^ (−0.686, −0.308)	96.59^***^ 25.36	199.25^***^ 11.78	<0.001	7 8	418 335	−3.103^***^ (−4.804, −1.403) −4.021^*^ (−7.561, −0.480)	−1.613^**^ (−2.716, −0.509) −0.353^*^ (−0.673, −0.032)	96.49^***^ 86.04^***^	155.60^***^ 86.41^***^	<0.001

*MD, mean difference; SMD, standardized mean difference; CI, confidence interval*.

* *p < 0.05*,

** *p < 0.01*,

*** *p < 0.001*.

The meta-analysis also showed that there was a significant improvement in number of awakenings (mean difference = −0.686, 95% CI −0.91 to −0.46, *p* < 0.001, *I*^2^ = 72.02) in the CBT intervention group compared with the control group, with large effect size (SMD = −1.18, 95% CI −2.10 to −0.26, *p* < 0.05).

The *Q*-test results were consistent with the *I*^2^ results in all analyses, indicating the same levels of significance of heterogenicity. The *p*-values between groups were all smaller than 0.001, suggesting that there was a significant difference between groups for each of the subgroup variables.

### Overall Effect of Face-to-Face CBT on Psychiatric Diseases

A total of 15 studies measured the effect of CBT on depression (31, 34–36, 43, 44, 46, 47, 49–52, 57–59) and the total number of participants in these studies was 753. We found that there was a significant improvement of depressive symptoms measured by depression scales (mean difference = −3.67, 95% CI −5.66 to −1.68, *p* < 0.001, *I*^2^ = 97.58) in CBT intervention groups compared with the control group, and there was a large effect size (SMD = −1.14, 95% CI −1.85 to −0.42, *p* < 0.01).

Subgroup analysis was also conducted on depression. The results for subgroup analysis on depression were presented in Table 5. The subgroup analysis showed that relapse prevention had greatest effect on depression. Studies that included relapse prevention reported a significantly greater improvement on depressive conditions (mean difference = −5.120, 95% CI −7.856 to −2.384, *p* < 0.001, *I*^2^ = 93.81) than studies that did not include relapse prevention (mean difference = −1.273, 95% CI −1.495 to −1.052, *p* < 0.001, *I*^2^ = 0.00). Other characteristics of the studies including drop-out rate <20%, number of sessions ≥6 sessions, duration of 1 session <1 h, length of intervention >6 weeks, individual delivered, using sleep drugs, and patients with co-morbid chronical diseases had significant effect on the reduction of depression. CBT intervention components including sleep hygiene, relaxation training, and basic sleep information had significant effect on the improvement of depression. The *Q*-test results were consistent with the *I*^2^ results, indicating the same levels of heterogenicity. The *p* between groups were all smaller than 0.001, suggesting that there was a significant difference between groups for each of the subgroup variables.

Anxiety was measured in eight studies (31, 35, 43, 47, 51, 52, 57, 59), and the total number of participants in these studies was 493. Compared with control groups, CBT intervention groups only reported a slight improvement in anxiety symptoms measured by anxiety scales (mean difference = −0.66, 95% CI −1.53 to 0.22, *p* > 0.05, *I*^2^ = 88.09), with moderate effect size (SMD = −0.62, 95% CI −1.55 to 0.32, *p* > 0.05). The *Q*-test result was consistent with the *I*^2^ result, indicating a high level of heterogenicity.

### Overall Effect of Face-to-Face CBT on Fatigue

Fatigue was reported as outcome measurement in six studies (34, 38, 48, 53, 55, 62), and the total number of participants in these studies was 426. The meta-analysis found that there was a significant reduction of daytime fatigue measured by different fatigue scales (mean difference = −4.74, 95% CI −9.34 to −0.14, *p* < 0.05, *I*^2^ = 99.94) in CBT intervention groups compared with the control group with a large effect size (SMD = −2.23, 95% CI −3.87 to −0.58, *p* < 0.01). The *Q*-test result was consistent with the *I*^2^ result, indicating a high level of heterogenicity.

### Overall Effect of Face-to-Face CBT on Quality of Life

The scores of SF 12 and SF 36 quality of life survey were reported in three studies (34, 42, 62). The scores of physical health section and mental health section were collected from all of the three studies. Patients in the CBT intervention groups showed a significant improvement in physical health measured by SF 12 and SF 36 survey (1.90, 95% CI 1.02 to 2.78, *p* < 0.001, *I*^2^ = 0.00), with a medium effect size (SMD = 0.42, 95% CI 0.08 to 0.76, *p* < 0.05). Mental health measured by SF 12 and SF 36 survey was also improved in CBT intervention group (4.95, 95% CI 1.01 to 8.90, *p* < 0.05, *I*^2^ = 80.07) compared with control group, with a large but not statistically significant effect size (SMD = 1.09, 95% CI −0.59 to 2.77, *p* > 0.05). The *Q*-test results were consistent with the *I*^2^ results, indicating the same levels of heterogenicity.

### Publication Bias

The results of the Egger's regression test for all of the research outcomes were summarized in Table 2. The funnel plots of the outcome variables were provided in Supplementary Figures 1, 2. A symmetric distribution of mean difference in all outcomes was observed upon the visual inspection of the funnel plots, indicating that there was no significant publication bias. The results of Egger's test of all variables had no significant results including Insomnia Severity Index (*t* = 1.05, 95% CI −0.89 to 2.69, *p* > 0.05), Pittsburgh Sleep Quality Index (*t* = 0.20, 95% CI −3.99 to 4.52, *p* > 0.05), total sleep time (*t* = −0.18, 95% CI −1.19 to 1.00, *p* > 0.05), sleep efficiency (*t* = 1.51, 95% CI −0.36 to 2.27, *p* > 0.05), sleep onset latency (*t* = −0.87, 95% CI −2.02 to 0.84, *p* > 0.05), wakening after sleep onset (*t* = −0.42, 95% CI −1.76 to 1.18, *p* > 0.05), number of awakenings (*t* = 0.98, 95% CI −1.22 to 2.71, *p* > 0.05), depression (*t* = 0.04, 95% CI −1.82 to 1.89, *p* > 0.05), anxiety (*t* = −0.86, 95% CI −1.89 to 0.90, *p* > 0.05), physical health (*t* = 1.00, 95% CI −9.78 to 11.45, *p* > 0.05), and mental health (*t* = −3.18, 95% CI −14.87 to 8.91, *p* > 0.05). These results suggested that there is no significant publication bias for these outcomes. The study by Pigeon et al. (53) was excluded in the analysis of fatigue because it provided a high publication bias for this outcome. The Egger's *t*-value for fatigue after excluding this study was −1.77 (95% CI −5.01 to 1.43, *p* > 0.05), which suggested that there is no significant publication bias for fatigue in the rest of the studies. Removing any single study in other outcomes did not change the overall results. Therefore, all of the included studies contributed to the overall publication bias results in all outcomes except fatigue.

## Discussion

This study included 31 randomized controlled trial studies to assess the effect of face-to-face delivered CBT on different health outcomes in patients with insomnia. The results from this meta-analysis study showed that face-to-face delivered CBT had a significant positive effect in improving all of the sleep outcomes (Insomnia Severity Index, Pittsburgh Sleep Quality Index, total sleep time, sleep efficiency, sleep onset latency, wakening after sleep onset, and number of awakenings), one of the psychiatric disease outcomes (depression), fatigue, and quality-of-life related physical and mental health. Face-to-face delivered CBT did not show significant effects on the other psychiatric disease outcome (anxiety).

### Efficacy of Face-to-Face CBT Intervention on Sleep Outcomes

The meta-analysis results were consistent with the findings from previous studies (12, 65), suggesting that face-to-face CBT had an overall significant effect in improving sleep quality and reducing insomnia symptoms. Previous studies (65, 66) mentioned that one of the major goals of CBT interventions on insomnia was to initially limit sleep opportunities at wrong time points in order to increase the pressure for sleep at right time points. Ultimately, the increased sleep drive would lead to an improvement of homeostatic regulation of sleep, and then right sleep opportunities at right time points. Variables such as sleep efficiency, sleep onset latency, and wake after sleep onset were related to homeostatic regulation of sleep, and these variables reported a relatively immediate improvement during the intervention, indicating an improvement of homeostatic regulation (65, 66). Other variables, including total sleep time and number of awakening, may show greater improvements over time as participants continue to practice the skills acquired during CBT intervention and gradually increase their opportunity for sleep (65, 66).

In randomized controlled trial studies, especially studies which were delivered in groups, frequent drop-out of participants may bring negative thoughts, such as untrust of the intervention and therapists, to other participants in the study. Participants would be less focused during the intervention sessions, and the overall effect of intervention would be reduced. Moreover, larger number of intervention sessions and longer duration of single sessions give the therapists a better chance to deliver the contents of the intervention in detail. Participants in these studies will get better understandings of the intervention contents and strategies and more likely to apply the strategies to their real life. We noticed that one of the included studies by Ellis (41) provided only one session of CBT intervention, but this study provided similar results on sleep outcomes compared with other studies which provided larger number of sessions. Considering this study was aimed to treat acute insomnia at early phase, further study was needed to confirm the effects of brief CBT on acute insomnia. In addition, longer intervention period could lead to the impatience of the participants and becoming unwilling to participate. Another important point is the form of delivery of the intervention. Patients in group-delivered CBT could discuss the contents of the interventions with peers, which promoted better understanding of the contents than patients who received CBT treatment alone. Also, the effect of face-to-face CBT was more significant when the patients had co-morbid psychiatric diseases or taking drugs such as hypnotics and antidepressants, which was consistent with the findings in previous study (67). A possible explanation for this result is that psychiatric diseases could be a main cause of insomnia. Although the studies included in this study all used CBT that was specially adapted for treating insomnia, some of the components such as cognitive therapy and relaxation training, still remained the functions of identifying and relieving anxious thoughts, which was effective in treating psychiatric disease symptoms. As a result, depressive or anxious symptoms were relieved due to CBT itself or other drug treatments. The relief of psychiatric disease symptoms led to a better mental health status, and therefore, insomnia was treated more effectively.

Sleep hygiene component was widely used as an individual treatment option in previous studies (68), which aimed to provide a better environment that is suitable for sleep. Although the effect of sleep hygiene alone on insomnia was very limited, a well-established bedroom environment is still advantageous when patients attempted to make behavioral changes (68). Consistent with the findings from previous study (69), relaxation training component was found to be effective. Relaxation training component taught the patients how to fall asleep more easily, establishing a positive mental condition toward insomnia, and patients with positive mental status are more determined to make behavioral changes and change their old sleep habits in order to improve sleep quality (69). As insomnia was a chronic condition, sustaining improvement is required after CBT intervention is finished. Therefore, relapse prevention is very important in CBT intervention program because the presence of relapse prevention component ensured the long-term effect of the intervention. In contrast, the rarely used components such as treatment rationale and basic sleep information did not seem to have great effects on sleep. These components only provided background information of disease and intervention to the patients, with limited effect on thoughts or behavioral changes. As the number of studies utilizing these components was limited, the effect of these components on sleep outcomes was uncertain.

### Efficacy of Face-to-Face CBT Intervention for Psychiatric Diseases

It has been reported in prior study that face-to-face CBT has a significant effect in improving depressive symptoms (65), which is similar to our findings. A possible explanation for the result would be face-to-face CBT intervention established a regularized sleep-wake cycle, and previous study mentioned that patients with a regularized sleep-wake cycle was more likely to experience a reduction in daytime symptoms, including depression and pain (65).

In previous studies, traditional cognitive behavioral showed an overall significant effect on anxiety (70, 71), which was quite different from our findings. However, all of the studies included in this study used CBT for insomnia (CBT-I), a specially adapted form of CBT targeting insomnia only. Different to the traditional CBT interventions, the contents in the CBT-I intervention focused on sleep problems and topics on anxiety were not discussed. On the other hand, only five studies reported anxiety in this study. Due to the limited number of studies, the results of meta-analysis on anxiety may not be representative.

### Efficacy of Face-to-Face CBT Intervention on Fatigue

It is recognized that the most direct consequence of poor sleep quality caused by insomnia is fatigue during the day (72). Face-to-face CBT has already shown a significant effect in improving sleep quality. It is plausible that patients receiving CBT intervention have developed a more efficient rest in bed, which led to an apparent reduction in daytime fatigue.

### Efficacy of Face-to-Face CBT Intervention on Quality of Life

The proposed mechanism for the improvement of physical and mental health is that face-to-face CBT intervention established a regularized sleep-wake cycle, which enabled the participants to get enough rest across the night. The adequate rest of the body would be helpful in reducing daytime symptoms such as depression, fatigue, and pain, leading to an overall increase in physical health. In this context, negative thoughts were rectified, and patients would have a more positive attitude toward life after the treatment. The improvement of both physical health and mental health contributed to the overall improvement of quality of life.

### Strength, Limitations, and Implications of Study

This study included a large number of high quality randomized controlled trial studies on the effectiveness of face-to-face delivered CBT in treating insomnia published in the last 22 years since the first RCT study (51) was published, and the results from subgroup analysis explained the contribution of specific subgroup variables to the overall effects. However, there are a number of limitations for the study. First, articles published in languages other than English were not included in this study. Second, although some of the research outcomes including Pittsburgh Sleep Quality Index, anxiety, fatigue, and scores of SF-12 and SF-36 health surveys were good indicators for sleep quality and health, they were reported in a very limited number of studies, making it hard to conclude if face-to-face CBT had a significant effect on those outcomes. Third, the effect of some CBT components was hard to measure because those components were presented in only one or two of the included studies, and we were not able to measure the heterogenicity and *p*-value between groups for those components. Therefore, further studies that include more trials with larger sample sizes, published in different languages, are needed to confirm the results of this study.

The using of face-to-face CBT when treating insomnia was highly recommended. When designing the CBT treatment plans, it is recommended that an appropriate design can include more than six sessions, duration of one session >1 h, length of intervention shorter than 6 months, and group delivered intervention. The retention of participants should be encouraged in the program, and the inclusion of the following components was recommended: sleep restriction, stimulus control, cognitive therapy sleep hygiene, relapse prevention, and relaxation training. Treatment rationale and basic sleep information was not recommended, but the inclusion of these components could still be considered if the circumstances permitted.

## Conclusion

In conclusion, face-to-face delivered CBT is effective in improving the sleep-related outcomes, including Insomnia Severity Index, Pittsburgh Sleep Quality Index, total sleep time, sleep efficiency, sleep onset latency, and wake after sleep onset in patients with insomnia. It was also found that face-to-face delivered CBT can improve other health outcomes in insomnia patients, including depression, fatigue, physical health, and mental health. Face-to-face delivered CBT is more effective when delivered through a larger number of sessions with longer duration of each session, and when delivered in groups. Apart from the most traditional components such as sleep restriction, stimulus control, and cognitive therapy, other components including sleep hygiene, relapse prevention, and relaxation training also showed promising effects and can be considered to add into study designs and treatment plans. More studies with larger sample sizes are required in further studies to confirm the findings.

## Data Availability Statement

The raw data supporting the conclusions of this article will be made available by the authors, without undue reservation.

## Author Contributions

DX: data collection, data analysis, and writing of original draft. JS: conceptualization, methodology, data collection, and reviewing and editing of writing. EC and SB: critically reviewing and editing of writing. All authors contributed to the article and approved the submitted version.

## Conflict of Interest

The authors declare that the research was conducted in the absence of any commercial or financial relationships that could be construed as a potential conflict of interest.

## Publisher's Note

All claims expressed in this article are solely those of the authors and do not necessarily represent those of their affiliated organizations, or those of the publisher, the editors and the reviewers. Any product that may be evaluated in this article, or claim that may be made by its manufacturer, is not guaranteed or endorsed by the publisher.

## Supplementary Material

Supplementary Figure 1 Funnel plots for sleep outcomes. **(A)** Insomnia Severity Index, **(B)** Pittsburgh Sleep Quality Index, **(C)** total sleep time, **(D)** sleep efficiency, **(E)** sleep onset latency, **(F)** wake after sleep onset.

Supplementary Figure 2 Funnel plots for sleep and health outcomes. **(A)** Number of awakenings, **(B)** depression, **(C)** anxiety, **(D)** fatigue, **(E)** Physical health, **(F)** mental health.
